# Myeloma as a Second Malignancy following AML: Is a Second Allo Equivalent to Auto?

**DOI:** 10.1155/2012/319530

**Published:** 2012-06-28

**Authors:** Sule Mine Bakanay, Esin Serbest, Klara Dalva, Isinsu Kuzu, Meral Beksac

**Affiliations:** ^1^Stem Cell Transplantation Unit, Hematology Department, Ankara University Medical School, Dikimevi, 06590 Ankara, Turkey; ^2^Tissue Typing Laboratory, Stem Cell Transplantation Unit, Hematology Department, Ankara University Medical School, Dikimevi, 06590 Ankara, Turkey; ^3^Department of Pathology, Ankara University Medical School, Dikimevi, 06590 Ankara, Turkey

## Abstract

We report a young male patient who developed plasma cell myeloma/plasmacytoma 11 years after having received an allogeneic hematopoietic cell transplantation for AML. The patient received a second transplantation from the same donor without immunosuppression and developed graft-versus-host disease (GVHD). Our observation has two aspects that warrant attention: first, insufficiency of long-term tolerance to prevent GVHD in the absence of immunosuppression and second, a stromal or genetic susceptibility to develop hematologic malignancies despite of a complete donor-type chimerism.

## 1. Introduction

 Allogeneic hematopoietic cell transplantation (HCT) is the only therapy successful in achieving cure for hematological malignancies. However, long-term survivors of allogeneic HCT face the risk of developing second malignancies, mainly the posttransplant lymphoproliferative diseases (PTLDs), secondary MDS/AML, and secondary solid malignancies [1]. Posttransplant lymphoproliferative disease presenting as plasma cell myeloma is extremely rare and represents a treatment challenge especially in presence of complete donor-type chimerism. 

## 2. Case Presentation

**Figure 1:**
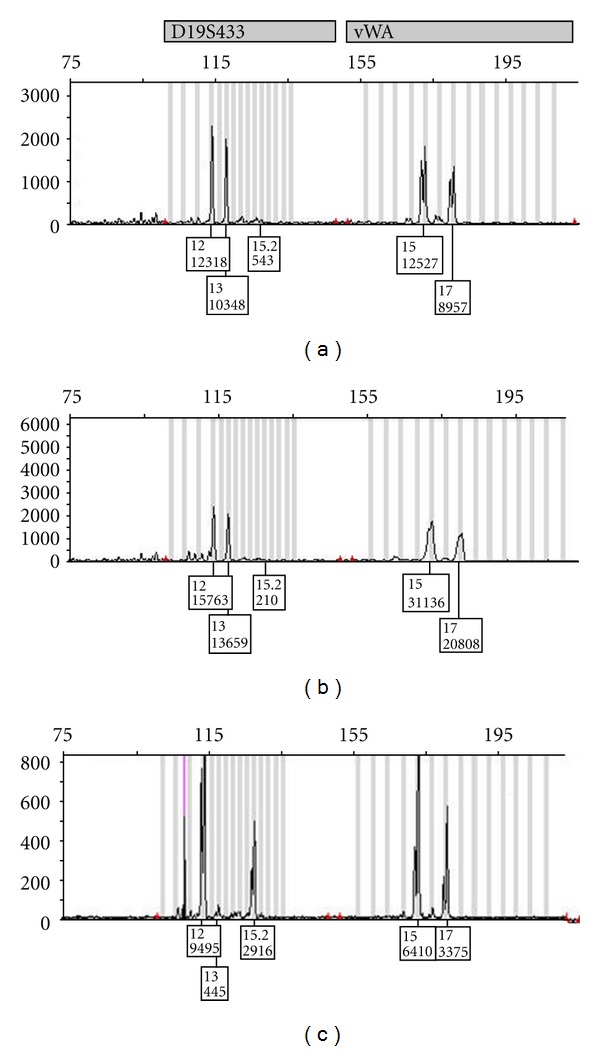
Chimerism analysis showing complete donor type in T-and non-T-cell lineages in the bone marrow (a, b) and mixed chimerism in the plasmacytoma (c).

 A 26-year-old male patient was diagnosed as AML-M2 in 1995. Following an induction regimen he achieved complete remission (CR) and received an allogeneic peripheral blood stem cell transplantation from his 37-year-old HLA identical brother in 1997. The conditioning regimen consisted of busulfan and cyclophosphamide, and the GVHD prophylaxis was done with cyclosporin and short-term methotrexate. He did not develop any acute or chronic GVHD and remained in CR with complete donor-type chimerism until 2008 when he was admitted to the neurosurgery clinic with back pain. The vertebral MRI revealed a tumor invading the 6th thoracic vertebrae causing pathological fracture. The tumor was completely excised, and the pathological evaluation was consistent with CD38^++^ and CD117^+^ atypical plasma cell infiltration with kappa monoclonality. He had IgG kappa monoclonal gammopathy on immune electrophoresis and an elevated erythrocyte sedimentation rate along with a mild anemia and normal renal function tests. Bone marrow (BM) examination revealed 9% plasma cells with CD38^+^CD138^+^CD19^−^CD56^−^CD44^+^CD28^−^CD20^−^ immunophenotype and kappa predominance. The cytogenetic study did not detect any abnormality. Bone marrow chimerism analysis was consistent with 100% donor type in both T-and non-T-cell lineages. Chimerism study was also done with the DNA extracted from the plasmacytoma and revealed 18% donor and 82% recipient cells (Figure 1). The presence of EBV could not be demonstrated in BM or plasmacytoma by in-situ hybridization for EBV early RNA as well as PCR analysis of EBV DNA. The donor was also found to be negative for signs of secretory paraproteinemia. The patient received 3000 cGy local radiotherapy to T5–T7 level of the spine and high-dose oral dexamethasone. After attaining a CR, it was decided to perform an allogeneic HCT without immunosuppression, instead of the general approach of autologous transplantation, since he had complete donor type of chimerism. Following a nonmyeloablative conditioning with fludarabine and melphalan a hematologic engraftment was achieved. The BM and blood examinations revealed CR for myeloma and a continued complete donor-type chimerism. The patient developed a grade 2 acute GVHD of skin which was controlled with steroids, but acute grade 3 gastrointestinal GVHD required the addition of cyclosporin. Later immunosuppression could be tapered off completely without causing rebound GVHD. He continued in CR until one year after transplantation when he developed progressive mucor sinusitis and infectious meningitis and eventually died.

## 3. Discussion

 Posttransplant lymphoproliferative diseases are a heterogenous group of disorders which develop after solid organ or hematopoietic stem cell transplantation as a result of immunosuppression. In contrast to the high incidence of posttransplant lymphomas, PTLDs manifesting as plasma cell myeloma/plasmacytoma are extremely rare. Most of the reported cases have developed after solid organ transplantation, mainly renal transplantation. The incidence of PTLD after allogeneic HCT is 1-2%, mostly diagnosed within the first year and strongly associated with EBV. On the other hand, EBV-negative PTLDs usually occur later [1–5]. The risk of PTLD after HCT increases significantly with T-cell depletion of the donor marrow and T-cell-targeted immunosuppression, unrelated or HLA mismatched grafts, severe acute/chronic GVHD, patient age older than 50 years, and receiving a second transplantation [6]. Transient monoclonal or oligoclonal gammopathies may occur early after transplantation and have been attributed to virus induced B-cell proliferation and insufficient T-cell surveillance. However, they are very rarely transformed into plasma cell neoplasms. Our patient did not possess any of the reported risk factors for PTLD development. The occurrence of plasma cell myeloma was also unrelated to EBV which is in contrast with most of the PTLD occuring after HCT. Although the patient's cellular immunity was not compromised for a long time, how the monoclonal plasma cells could evade the immune surveillance and plasma cell myeloma developed requires explanation. There is always the possibility that this might be a conventional de novo plasma cell myeloma/plasmacytoma because myeloma can also be observed in young patients. In the literature, we could find only two reports of plasma cell myeloma/plasmacytoma developing after allogeneic HCT [7, 8]. 

 Although the patient was in complete donor-type chimera when he was retransplanted from the same donor 11 years later with fresh peripheral blood stem cells, he still developed GVHD and required immunosuppression. We were not able to find any reports to guide us in our decision making regarding the immunosuppression strategy at the time of retransplantation. Spontaneous occurrance of autologous GVHD in myeloma patients undergoing autologous HCT has been recently reported [9]. On the other hand, additional lymphocytes in the peripheral blood stem cell product infused from the same donor might have behaved like a donor lymphocyte infusion. This observation challenges the concept of long-term immune tolerance. Recipient antigen presenting cells, which are known to play a critical role in initiating GVHD, were in a tolerant status until the second transplantation [10]. Moreover, the BM microenvironment could be an inciting factor. Still, we cannot exclude the changes in donor and recipient immune repertoire and immunogenicity acquired during the 11 years following the first transplantation which seems to have destroyed the donor versus recipient tolerance while inducing new antigenic targets.
